# A comprehensive review of bacterial and hemoparasitic diseases in the water buffalo

**DOI:** 10.3389/fvets.2026.1812517

**Published:** 2026-06-04

**Authors:** Hugo B. Barrios-García, Verónica Carvajal-de la Fuente, Jorge Alva-Pérez, Belkis Corona-González, Dasiel Obregón Alvarez, Dora Romero-Salas, Daniel Mota-Rojas, Octavio Merino-Charrez, Julio Martínez-Burnes

**Affiliations:** 1 Animal Health Group, Facultad de Medicina Veterinaria y Zootecnia, Universidad Autónoma de Tamaulipas, Victoria, Tamaulipas, Mexico; 2 Laboratorio de Hemoparásitos, Centro Nacional de Sanidad Agropecuaria (CENSA), La Habana, Cuba; 3 School of Environmental Sciences, University of Guelph, Guelph, ON, Canada; 4 Laboratorio de Parasitología, Facultad de Medicina Veterinaria y Zootecnia, Universidad Veracruzana, Veracruz, Mexico; 5 Neurophysiology, Behavior and Animal Welfare Assessment, DPAA, Universidad Autónoma Metropolitana (UAM), Mexico City, Mexico

**Keywords:** bacterial diseases, *Bubalus bubalis*, health, hemoparasites, parasitic diseases, water buffalo

## Abstract

Water buffalo exhibit low mortality rates and high resistance to pathogens. They are less susceptible to developing diseases common in other bovids; however, they are susceptible to various bacterial agents and hemoparasites. Although buffalo are relatively resistant to the clinical form of many diseases, they can serve as reservoirs for various pathogens, facilitating their spread to other susceptible species, which is particularly relevant in a One Health perspective. This review compiles information on economically important infectious diseases affecting buffalo herds, including bacterial infections (brucellosis, tuberculosis, paratuberculosis, leptospirosis, salmonellosis, etc.), vector-borne diseases (anaplasmosis, babesiosis, theileriosis, trypanosomiasis), neosporosis, and toxoplasmosis, among others. To this end, a systematic review was conducted, analyzing 180 articles from scientific databases such as Web of Science, PubMed, Google Scholar, and SciELO. The inclusion criteria were studies focused on different bacterial and parasitic etiological agents reported to affect water buffalo. The review findings indicate epidemiological trends of increasing involvement of water buffalo in the circulation of infectious diseases in mixed livestock systems. Water buffalo can act as a reservoirs and sources of interspecific transmission, especially given their due to the frequency of subclinical infections and proximity to cattle. These findings highlight the need to include this species in surveillance and health management programs. However, gaps remain in research on specific epidemiology and there is a lack of systematic studies. The increasing global expansion of buffalo production and the associated risks to animal and public health underscore the importance of conducting evidence-based studies to strengthen disease control and prevention strategies.

## 1 Introduction

The water buffalo can adapt to adverse environmental conditions, especially in a humid tropical climate (1, 2) where high temperatures and poorly drained soils predominate. This has favored its expansion throughout the American continent since its recent introduction in the Caribbean and Brazil in the latter part of the 19th century (3). However, the environmental conditions of humidity and heat are ideal for the growth of microorganisms, which can adversely affect the health of this animal species (4–7).

Generally speaking, the water buffalo has low mortality rates and high resistance to pathogens, making it less susceptible to developing diseases commonly observed in other bovids (8). However, they are sensitive to various infectious agents affecting traditional cattle, which can cause bacterial and parasitic diseases of veterinary relevance, including tuberculosis, paratuberculosis, brucellosis, leptospirosis, and hemorrhagic septicemia. Furthermore, other reports indicate enteritis caused by *E. coli* and *Salmonella* spp., as well as cases of clinical and subclinical mastitis caused by various pathogens (9). On the other hand, although buffaloes are resistant to the clinical form of several diseases, they can serve as reservoirs of multiple pathogens, facilitating the contagion of other susceptible species, mostly cattle of the *Bos* genus (10), including hemoparasites and other protozoa reported in buffaloes, such as *Neospora caninum* and *Toxoplasma gondii*.

Despite the growing number of reports on infectious agents affecting water buffalo (*Bubalus bubalis*), current knowledge remains fragmented, focused on specific pathogens or geographically limited, and is frequently extrapolated from cattle without fully considering the biological and epidemiological characteristics of this species. This limits our understanding of the water buffalo’s role in the maintenance, transmission, and modulation of bacterial and hemoparasitic diseases, especially in regions where buffalo and cattle coexist, particularly on expanding livestock operations in Africa and South America. Given the rapid expansion of buffalo production systems, their close interaction with traditional livestock farming, and the relevance of several buffalo-associated pathogens to animal and public health, a One Health framework is essential to understand the interactions between animals, humans, and their shared environments. There is an urgent need to integrate and analyze the available evidence. This review contributes by compiling and synthesizing recent findings on bacterial, hemoparasitic, and protozoan diseases affecting water buffalo, highlighting knowledge gaps and providing an updated epidemiological perspective that supports surveillance and health control strategies.

## 2 Materials and methods

### 2.1 Systematic review

A literature search was conducted across scientific databases, including Google Scholar, Web of Science, PubMed, and SciELO. Combinations of keywords such as “Water buffalo,” “*Bubalus bubalis*,” “pathogenic bacteria,” and “hemotropic pathogens” were used to retrieve articles. Articles providing specific data on bacterial diseases, brucellosis, tuberculosis, paratuberculosis, anthrax, salmonellosis and colibacillosis, leptospirosis, hemorrhagic septicemia, anaplasmosis, babesiosis, theileriosis, trypanosomiasis, neosporosis, and toxoplasmosis were included. No restrictions were applied regarding the publication date of the literature review. The final search yielded a database of 180 articles according to predefined criteria. Of these publications, at least 90 (50%) were published within the last decade.

## 3 Bacterial diseases

### 3.1 Brucellosis

Given its worldwide zoonotic impact, special attention is paid to brucellosis, a transmissible and infectious disease caused by the gram-negative bacterium Brucella (11, 12). Brucellae are intracellular pathogens in lymphoreticular tissues and mainly cause reproductive losses in natural hosts. *Brucella* uses multiple molecular mechanisms to promote pathogenesis (13). Brucellosis in water buffalo (*Bubalus bubalis*) is mainly attributed to *Brucella abortus* (14), but there are also reports of *Brucella melitensis* (15). In buffaloes, biotypes 1, 3, and 6 of *B. abortus* have been identified, and biotype 7 less frequently; from *B. melitensis,* only biotypes 1 and 2 have been identified (12, 15, 16). *B. abortus* biotype 1 is the most frequently isolated in buffaloes (16, 17). Water buffaloes are generally more resistant to brucellosis than cattle (*Bos*). In challenge tests, a higher bacterial concentration was required to induce seroconversion detected by rose Bengal plate tests (RBPT), suggesting a higher infective dose in water buffalo (18).

Epidemiology. Brucellosis has been reported in buffaloes in many countries, including Africa, Central Asia, southern Europe, and even the Americas (19), with a global estimated seroprevalence of 9.7% in 2021 (20). In 2024, in the Haryana state of India, the observed seroprevalence was 8.25 and 7.5% for RBPT and iELISA, respectively (21). In Iran, a DNA survey of buffalo milk samples detected up to 46% of positivity. The DNA identified was mainly from *B. abortus,* and a minor proportion of *B. melitensis* (22). An analysis conducted in Pakistan revealed a prevalence of 26.08% by bacteriological isolation in buffalo milk and seropositivity of 13.9 and 15.4% by Rose Bengal Plate Test (RBPT) and iELISA, respectively (23). In Tanzania, a prevalence of 7.9% was reported (24). In Europe, especially in Italy, a high prevalence of brucellosis has been reported (11). In America, brucellosis is also reported in buffaloes in Argentina, Brazil, Costa Rica, Colombia, and Mexico (25–27). In Colombia, in 2022, a cross-sectional study demonstrated that seroprevalence in Murrah buffaloes was 2.08% using RBPT and competitive ELISA (cELISA) (28). In Argentina and Brazil, *B. abortus* has been confirmed by isolation (29, 30). Using RBPT and ELISA, Colombia has reported seroprevalences of 12 and 3% for *B. abortus, respectively,* (31). In Venezuela, a seroprevalence of 51.25% with Rose Bengal have been reported (32). The first report on Brucellosis in Mexico’s water buffalo was published in 2012. Serological studies using the Bengal Rose Test and Rivanol were conducted on 565 water buffaloes from three farms. Global seroprevalence was 13% with the Rose Bengal and 7% with Rivanol. In two livestock production units, buffalo coexisted with cattle, sharing grazing areas and water sources; therefore, diagnostic tests for brucellosis were performed on 75 specimens, yielding negative results in all cases; therefore, it is a priority to strengthen community awareness strategies and to develop additional epidemiological studies that characterize the dynamics of disease transmission in wildlife, livestock, and human populations within the area of interest (19, 33). Also in Mexico, a seroprevalence of 17.7% against *B. abortus* was identified by ELISA in water buffaloes from Veracruz and Tabasco (34, 35). Recently, in Mexico a cross-sectional epidemiological study in water buffaloes reported a *Brucella* seroprevalence of 4 and 3%, with RBPT and Rivanol tests, respectively. This study included three different southern states of the country, with similar agroecological conditions (36).

Transmission of *B. abortus* is through the ingestion of contaminated food or secretions from aborted fetuses and genitals. The bacterial proliferation *in utero* causes necrosis and injury of both fetal and maternal placental membranes, leading to fetal death and expulsion (13). A massive shedding of brucellae begins after the abortion and may continue for 15 days. Some infected traditional bovines become carriers of brucellae and excrete them intermittently for many years. In newborns, the infection can originate in the uterus or occur when calves from healthy mothers are fed colostrum or milk from infected females. Infection can also occur through the semen of infected bulls during copulation or through skin lesions. Sheep, goats, and wild animals play a key role in the transmission of the disease among buffaloes (37). The clinical signs of brucellosis include abortion, retained placenta, and impaired fertility, and are related to susceptibility or resistance of individuals. Abortion can occur in the final stage of pregnancy, accompanied by placental retention and catarrhal-type metritis. In bulls, epididymitis and orchitis can occur, semen quality can be affected, and they can remain carriers, in addition to synovitis in affected joints. The economic loss lies in the slaughter of infected animals in developed countries, in abortions, and in low milk production in developing countries (34, 37). Table 1 shows the reported prevalence of brucellosis worldwide by region.

**Table 1:** Worldwide distribution and reported prevalence of major infectious diseases in the water buffalo (*Bubalus bubalis*).

Etiological agent/disease	Region (by continents or subregions)	Reported prevalence	References
*Brucella abortus*/Brucellosis	Africa (Tanzania)	7.9%	(24)
	South Asia (Pakistan) India	26.08% isolation in milk; 13.9% RBPT; 15.4% i-ELISA 8.25% RBPT 7.5% iELISA	(23, 28)
	Europe (Italy)	High prevalence (not quantified)	(11)
	South America (Argentina, Brazil) Brazil	Presence confirmed by isolation 79.31% (iELISA) 38% (rtPCR)	(29, 30, 154, 155)
	North America (Mexico)	13% RB; 7% Rivanol-17.7% ELISA	(33, 34, 35)
	South America (Colombia) (Colombia)	12%RBPT; 3% ELISA 2.08% (RBPT, cELISA)	(28, 30)
	Caribbean (Venezuela)	51.25% Rose Bengal	(32)
*Mycobacterium bovis*/Tuberculosis	Africa (South Africa)	17.6%	(51)
	Africa (Tanzania, Uganda)	Reported presence	(14, 50)
	Oceania (Australia)	Reported presence	(49)
	Asia (Indonesia, Thailand)	Reported presence	(49, 50)
	Europe (Italy)	Presence (INF-y)	(48)
	South America (Brazil)	4.16%	(53)
	South America (Argentina)	First isolation reported	(52)
	Caribbean (Cuba)	Presence (experimental tuberculin)	(53, 56)
*Mycobacterium avium*, subsp. Paratuberculosis	South Asia (India)	68.2–71.8%; 28.6% 22%	(64, 65, 66)
	South America (Brazil)	Reported presence	(67, 68)
	South America (Colombia)	Reported presence	(69)
	North America (Mexico)	27%	(70, 71)
*Bacillus anthracis*/Anthrax	Africa	17% mortality in outbreaks	(79)
	Asia/America	Occasional presence (not quantified)	(80)
*Salmonella* spp. *E. coli* (gastrointestinal illnesses)	South Asia (Pakistan)	Leading cause of mortality in calves	(93)
	Tropical Regions (global)	Up to 70% mortality from diarrhea in calves	(87)
*Leptospira interrogans*/Leptospirosis	North America (Mexico, Veracruz)	34.7%	(104)
	Northeast India	25%	(103)
	South America (Brazil)	70.6%; PCR 2.3%	(97, 99)
	South America (Argentina)	22.2%	(102)
	Asia (Thailand)	30.5%	(98)
	Middle East (Iran)	43.3%	(106)
*Pasteurella multocida*/Hemorrhagic septicemia	Africa and Asia	High mortality (up to 100%)	(114)
*Babesia bovis/Babesia bigemina*	Latin America (Brazil, Argentina, Mexico, Cuba)	Variable presence; less clinically significant than in cattle	(117, 118, 136)
*Anaplasma marginale*	Caribbean and Latin America	49–60% seroprevalence; low molecular detection	(118, 130)
*Theileria buffeli*/*Orientalis*	Asia, Africa and Europe Southeast Asia	Reported presence	(137, 142, 143, 144, 145, 146, 178)
	South America (Brazil)	Sporadic molecular presence	(147)
*Trypanosoma vivax*	South America and the Caribbean	Up to 59.6% in cattle; 44.3% in buffalo	(147)
	Venezuela	23–40%; clinical outbreaks	(153, 156)
*Trypanosoma evansi*	Asia (Vietnam, Indonesia)	22% seropositive; endemic disease	(150, 151)
*Neospora caninum*	North America (Mexico, Veracruz)	44.8–71.6%	(169, 170)
	Europe (Italy)	34.6%	(163)
	South America (Brazil, Argentina)	64%	(164, 167)
	Southwest Asia (Turkey)	12%	(166)
	Northeast Africa (Egypt)	58.8%	(165)
*Toxoplasma gondii*	North America (Mexico)	High seroprevalence (48.7%)	(173)
	South America (Brazil, Argentina)	25.4–35.5%	(102, 174)
	Caribbean (Cuba)	55.3%	(176)
	Southwest Asia (Turkey)	21%	(166)

In water buffalo, the same control strategy has been followed as for cattle (*Bos*); for more than two decades, the immune response induced by the vaccine in buffaloes with the *Brucella abortus* strain 19 has been evaluated (38, 39). In Egypt, the “test and slaughter” policy was applied in dairy herds infected with *Brucella* (40); another action is the quarantine of infected herds. The Italian government has carried out an eradication program since 1994, which has allowed the decrease in the prevalence and geographical distribution of brucellosis (41), and the European Commission approved the use of the vaccine RB51 strain of *B. abortus* for water buffalo (RB51) since 2007 (11). Although a vaccination program began in 2007 in Iraq, it needs to be better implemented (42). In Mexico, pregnant water buffalo females have been vaccinated with the RB51 strain, and the calves have been reported not to present seroconversion; the females do not present abortions either, and there is no possibility of persistent infections. Revaccination with the same vaccine does not alter the conventional serological status, indicating that multiple vaccinations in normal adults and pregnant females can increase immunity without altering the serological status (43, 44).

Overall, the evidence indicates that brucellosis in water buffalo presents epidemiological and host-response differences compared with cattle, limiting the direct transferability of bovine-based control strategies. Within a One Health framework, the wide variability in reported prevalence highlights the influence of regional management practices and mixed-species systems, underscoring the need for buffalo-specific surveillance and control approaches.

### 3.2 Tuberculosis

Tuberculosis is an important disease, given its economic and zoonotic impact (45). It is a chronic, contagious, infectious disease, caused by *Mycobacterium*, that affects multiple host mammal species, in wild fauna and animals in captivity. It is a chronic, contagious, infectious disease caused by *Mycobacterium* that affects multiple host mammal species, both wild and captive. It has been recognized worldwide for many years (13, 46, 47). *Mycobacterium bovis* is the main source of infection in cattle of the genus *Bos* and in water buffalo. It poses a serious threat to human health, as it is a zoonotic disease and the leading cause of infection in developing countries (48).

The first reports of *M. bovis* isolation in water buffaloes were in 1968 in Tanzania (14); later in Australia and Indonesia (49), and in Uganda and Thailand in 1995 (50). More recently, a study conducted in South Africa found that the prevalence of *M. bovis* was 17.6% in free-living buffalo (51). In Europe, especially in Italy, it has also been reported using the INF-*γ* test (48). *M. bovis* has also been reported in water buffalo in the Americas; in Argentina, in 2006, the first isolation was reported (52); in Brazil, with a prevalence of 4.16% (53), and a new clade of *M. bovis* strain (54). In Colombia, a variable prevalence across regions and with different techniques was reported (55). It has also been reported and studied in Cuba, using an experimental tuberculin test to evaluate a practical, inexpensive diagnostic method that, apparently, has been satisfactory; however, much remains to be evaluated since the thickness of buffalo skin differs from that of cattle (53, 56).

*M. bovis* induces a chronic disease that lasts for months or years; affected animals are generally asymptomatic or may present characteristic signs such as progressive emaciation, coughing, dyspnea, lymphadenomegaly, and decreased production; mastitis may be present. It produces caseous lesions in lymph nodes, mainly in the lungs, mediastinal and mesenteric lymph nodes, liver and pleura, until forming areas of caseous necrosis undergoing advanced dystrophic calcification processes that are usually yellow in bovines (*Bos*) and white in buffaloes. The lesions in buffaloes are lighter in tone and show a lower degree of calcification than those in cattle (57). Caseous necrosis suggests tuberculosis; to verify whether a herd is infected, the tuberculin test is used as a practical, inexpensive diagnostic tool (53, 58). Nevertheless, for a definitive diagnosis, it must be supported by histopathology, bacteriological isolation (59), molecular (PCR) or INF-*γ* (48) However, some infected animals do not show detectable evidence. Hence an alternative such as humoral response-based testing is a useful tool for identifying infections that go unnoticed with traditional methods (60).

Tuberculosis control is based on eradication, first, diagnosing positive cases using tuberculin and eliminating infected animals through slaughter; and, as sanitary measures, the physical separation of animals for raising offspring (48). For countries where buffaloes are newly introduced, this species’ induction risk should be considered.

Taken together, the available evidence suggests that tuberculosis in water buffalo is characterized by diagnostic and pathological particularities that limit the effectiveness of cattle-based detection and control programs. Within a One Health framework, the wide variation in reported prevalence and diagnostic performance across regions underscores the need to adapt surveillance protocols and testing strategies to buffalo-specific biological traits, especially in mixed-species and newly established production systems. Table 1 shows the reported prevalence of tuberculosis by regions worldwide.

### 3.3 Paratuberculosis

Another pathogen involved in water buffalo diseases is *Mycobacterium avium paratuberculosis* subspecies (MAP) (61, 62), a member of the genus acid-alcohol-resistant bacteria and is responsible for paratuberculosis in animals or Johne’s disease in humans or bovine chronic enteritis [56]. It has been reported in outbreaks or endemic in several countries in productive animals and wildlife. Epidemiology. Paratuberculosis is an infectious, contagious, chronic disease with worldwide distribution associated with domestic and wild ruminant (13). It has been reported in water buffalo in India; in a study evaluating two serological tests, the MC-ELISA and the i-ELISA the seroprevalence was 68.2 and 71.8%, respectively, (63, 64). In another study in India, 28.6% seroprevalence was obtained by ELISA (65). Another study strengthens this, reporting 22% seroprevalence in Madhya Pradesh, India (66). In America, it has been reported in Brazil (67, 68); in Colombia (69), and in Mexico with a seroprevalence of 27% (70, 71).

Mycobacteria enter orally through food contaminated with feces from infected animals. Infection is common in young animals, followed by a prolonged incubation period with intermittent fecal excretion of small numbers of microorganisms. When bacteria are present in high numbers, extensive intestinal lesions develop, causing clinical disease and colonizing the mesenteric and ileocecal lymph nodes. The mycobacteria are shed in feces, where they can contaminate the environment (13). Lesions in water buffaloes include thickening of the intestinal mucosa and inflammation of mesenteric lymph nodes, with mild to moderate granulomatous inflammation (72). The definitive diagnosis is by bacteriological isolation; however, this is prolonged, so options such as PCR or ELISA are available (65).

Regarding the control, in a study of 48 nations, paratuberculosis was very common in cattle (*Bos*) and buffalo. In nearly half of the countries, more than 20% of herds and flocks had MAP infection, and in most (60%), there were voluntary control programs (73). Most countries’ general strategy is to identify and remove sick and/or subclinical infected animals (68, 74).

Overall, the high seroprevalence and prolonged subclinical course of MAP infection in water buffalo suggest substantial underdiagnosis and sustained environmental transmission. These characteristics limit the effectiveness of cattle-derived control approaches and support the need for buffalo-specific diagnostic and surveillance strategies. Table 1 shows the reported prevalence of paratuberculosis worldwide by region.

### 3.4 Anthrax

Anthrax is an infectious disease caused by the sporulated Gram-positive bacterium *Bacillus anthracis*, which induces acute and chronic septicemia affecting numerous domestic and wild animals, as well as humans (75, 76). The disease occurs worldwide and is one of the most important zoonoses (13, 77, 78).

In water buffalo, recurrent anthrax outbreaks have been reported between 2014 and 2017 in Africa, where 745 out of 4,500 buffaloes were affected, indicating a mortality rate of 17% (79). Nevertheless, there are few official reports of this disease, although traditional cattle are generally more susceptible than buffaloes (80).

Anthrax can be transmitted through infected animals’ blood, meat, and hides, as well as by inhalation or ingestion of spores through contaminated food, forages, water, or corpses. Outbreaks are more frequent in warm, humid weather (80, 81). It is an infectious process of febrile and septicemic nature, characterized by sustained hyperthermia and the possible evolution to meningitis, which may be in a few days, be followed by an acute disease in several animal species. However, in buffaloes, there are acute and hyperacute forms. It induces localized ulcers and scabs. Inhaled *Bacillus anthracis* causes fulminant pneumonia (82).

Anthrax affecting the intestine typically manifests as acute gastroenteritis, with symptoms such as nausea, vomiting, and bloody diarrhea; splenomegaly is common. Bleeding through natural orifices is a frequent event and is often associated with high mortality. The clinical evolution in 48 h includes fever of 42 °C, depressive state, rapid and profound breathing, as well as congestion of the mucous membranes, accompanied by hemorrhagic zones. Milk may have a reddish color due to the presence of blood; diarrhea and swelling of the tongue, throat, sternum, and perineum may occur. Death is sudden, following convulsions and collapse, with no other signs except bleeding from the nostrils, anus, and mouth (80, 83).

Anthrax in water buffalo is managed in much the same way as in cattle (*Bos*); however, the physiological requirements of water buffalo, especially young specimens, are not the same as those of cattle. Buffalo wallowing behavior of increases calves’ exposure with this bacterium, thereby increasing the frequency of the disease compared with traditional cattle (84). The spore vaccine is effective and provides protection for one year; its use can be beneficial in high-risk situations. After an outbreak, it is recommended to administer it annually for at least 3 years (85). In cases with the presence of the pathogen, necropsy or slaughter of the animal should be avoided to prevent sporulation of the vegetative forms of *B. anthracis* upon contact with oxygen. The carcass must be destroyed, preferably cremated in the same place, and the ashes must be buried. If cremation is not possible, the cadaver must be buried at a depth that ensures at least one meter of earth above it, to prevent it from being dug up by animals (81, 86).

In water buffalo, anthrax risk in modulated by species-specific behavior and environmental exposure, which may potentially reduce the effectiveness of cattle-based control measures. These factors, and underreporting, support the need for buffalo-adapted surveillance and prevention strategies. Table 1 shows the reported prevalence of anthrax worldwide by region.

### 3.5 Bacterial gastrointestinal diseases

Salmonellosis and colibacillosis are the main bacterial gastrointestinal diseases reported in buffaloes, mainly in calves from 6 to 12 months. In dairy herds, it has been reported that up to 70% of diarrhea-related mortality in water buffalo calves is due to *Salmonella* spp., and *S. Thyphimurium* the main serovar identified (87). *Escherichia coli* and *Salmonella* spp. are Gram-negative Enterobacteriaceae that have numerous virulence factors. Although *E. coli* has been considered an inhabitant of the colonic microbiota in domestic species, *Salmonella* spp. is always associated with enteric or systemic problems rather than the intestinal microbiota (88–90). In addition to *Salmonella* spp., *Escherichia coli* (enterotoxigenic, ETEC, enterohaemorrhagic, EHEC, and necrotoxigenic, NTEC) and *Clostridium perfringens* have also been isolated from young animals. Other non-bacterial agents involved in diarrhea in *B. bubalis* calves are *Cryptosporidium* and Rotavirus (87, 91, 92). Similar results have been found by Zaman et al. (93), who report that one of the leading causes of death of water buffalo calves in Pakistan is diarrhea and pneumoenteritis, mainly due to *E. coli* and *Salmonella*. The main sources of transmission and contagion are excretion in the feces of asymptomatic carriers, as well as fomites and mechanical spreading.

In addition to profuse diarrhea, enteritis, pyrexia, and dehydration, severe cases may also involve systemic infection (joint and central nervous system infection). These signs are similar to those reported in cattle (*Bos*); however, *Bubalus bubalis* is more susceptible to fever, due to the low proportion of sweat glands. The changes in acute-phase proteins induced by *Salmonella* sp. infection (increases in fibrinogen, haptoglobin, and ceruloplasmin, and a decrease in transferrin) are consistent with clinical signs (94). The complete blood count and leukocyte changes (polycythemia and leukopenia) are also comparable to those observed in other ruminants (94).

*E. coli* has been isolated from fecal samples with different antibiotic resistance genes (92, 95), which represents a significant risk in the control of these pathogens. This reflects an incorrect use of antibiotic therapy, although it is not exclusive to water buffalo production. Regarding the risk to public health, *B. bubalis* is a carrier of *E. coli* O157: H7 (92, 96), with the potential to intermittently excrete and spread in crops or dairy products.

Overall, the high mortality associated with enteric infections in buffalo calves, together with antimicrobial resistance and zoonotic potential (e.g., *E. coli* O157: H7), highlights these pathogens as a critical One Health concern. The role of asymptomatic carriers and environmental contamination underscores the need for integrated animal-human-environment surveillance and control strategies adapted to buffalo production systems. Table 1 shows the reported prevalences worldwide of *Salmonella* spp. and *E. coli*. by region.

### 3.6 Leptospirosis

Because water buffaloes can adapt to humid tropical and subtropical climates, the risk of infection with pathogenic *Leptospira* serovars is high. The bacteria of the genus *Leptospira* are Gram-negative, comprises more than 260 serovars; the species *L. interrogans* is the most significant pathogenic species worldwide. It is more commonly found in humid environments and has a wide host range, including cattle, dogs, pigs, ruminants, and man (97). Water buffalo can be up to three times more susceptible to infection than traditional cattle, and they can share it with different serovars if they live closely together (98, 99). The main transmission routes are oral and skin abrasions, with venereal transmission not ruled out. In *B. bubalis*, *Leptospira* spp. infects the renal tissue, and the sexual organs, allowing it to be excreted in both urine and semen, as well as in milk and placental fluids (100, 101).

Given its significant capacity to cause human disease (bacterial shedding in the urine), seroepidemiological surveillance of *Leptospira* has been conducted in several countries. Water buffalo are carriers and shedders, as some *L. interrogans* serovars do not cause disease or produce inapparent clinical signs (102). In a study conducted in Northeast India, the seroprevalence was 25% (103). In Veracruz, Mexico, Romero-Salas et al. (104) found a seroprevalence of 34.7%, with the Muenchen serovar being the most significant. In the northwestern region of Brazil, a seroprevalence of 70.6% was found; the most frequent serovars are Pomona, Butembo, and Icterohaemorrhagiae (105). In Argentina, Konrad et al. (102) reported a seroprevalence of 22.2%, with Pomona, Canicola, and Grippotyphosa as the predominant serovars. In Thailand, in a serological study carried out by Suwancharoen et al. (98), they found a prevalence of 30.5%, higher than that observed in bovines (9.9%), and the prevalent serovars were Ranarum, Sejroe, and Mini. In Iran, a seroprevalence of 43.3% was observed, with the Hardjo serovar showing the highest positivity rate (56.2%). Of the serological microagglutination test (MAT) cases, only 15.6% were positive for PCR (106). On the other hand, under alluvial plain conditions, Guedes et al. (107) recently found evidence of infection by PCR in only 2.3% of a region of the Amazon in Brazil. The identified species were *L. interrogans* and *L. borgpetersenii*. These results indicate that serology helps determine the infection status among positive reactors, while PCR determines the active dissemination of these bacteria.

The pathogenesis of leptospirosis in *Bubalus bubalis* is partially known; however, reproductive problems similar to those of bovines (*Bos*) are likely to occur, such as abortions, infertility, and decreased milk production (107, 108). Susceptibility to infection is also age-dependent, with more MAT test-positive animals observed in adults in Italy (109) and Thailand (98). *L. interrogans* causes kidney lesions, such as interstitial nephritis, acute tubular necrosis, pyelonephritis, glomerulonephritis, renal fibrosis, and hydronephrosis (106).

The control of leptospirosis in water buffaloes has not been sufficiently reported. There are few studies on the species and pathogenic serovars of Leptospira, and it is necessary to better understand the natural evolution of the disease (106, 107). It is likely that in *B. bubalis*, the chronic form of the disease develops, causing them to become asymptomatic carriers that intermittently shed these bacteria. Since *Leptospira* species tend to be maintained in specific maintenance hosts, vaccination must be based on the species or serogroups isolated from infected buffaloes, as in traditional cattle (98, 110).

Overall, the high exposure risk, frequent subclinical carriage, and intermittent shedding of pathogenic Leptospira in water buffalo position this species as a key reservoir at the animal-human-environment interface. Within a One Health framework, the marked discrepancies between serological and molecular detection emphasize the need for integrated surveillance strategies and buffalo-adapted vaccination programs to reduce zoonotic and environmental transmission. Table 1 shows the reported prevalence of Leptospirosis worldwide by region.

### 3.7 Hemorrhagic septicemia

Pasteurellosis, produced by *Pasteurella multocida* subspecies multocida in cattle (Bos) and water buffaloes, is known as Hemorrhagic Septicemia. *P. multocida* is a Gram-negative bacillus that presents a capsule, one of the main virulence mechanisms. This disease is significant in *B. bubalis*, as it is a reservoir. In Vietnam, where *Pasteurella multocida* is reported as one of the main causes of hemorrhagic septicemia, a study by Nguyen in 2025 goes beyond the typical bacterial isolation the published molecular data include lipopolysaccharide capsular typing, virulence-associated genes, and antimicrobial resistance genes, being one of the first studies to characterize the isolates (111);. Several serotypes of *P. multocida* are associated with disease (B:2, A;1, A;3, B:3, B:4, E:2, and B:1), the most common being serotypes B:2 and E:2 (Carter-Heddleston classification system) (112, 113). Hemorrhagic septicemia is present mainly in Africa and Asia, but not in America. However, serotypes B:2 and E:2 have been reported to be isolated from deer in the United States. In Mexico, it is an exotic disease of obligatory reporting. Although the disease occurs frequently in cattle and water buffalo, the latter is more severe (100% morbidity and mortality) (114). In endemic regions, young animals are more susceptible than adults, up to 2 years of age. In epizootic outbreaks, it is observed in individuals of all ages. *P. multocida* subsp. *multocida* is mainly transmitted by aerosols (the bacterium finds its ecological niche in the oropharyngeal region), penetrating the oral and respiratory mucosa. Humid climates favor spreading, and stress tends to infect susceptible animals; more cases are detected in the rainy season. The incubation period is up to 5 days, with a scarce chance of recovery. Recovered individuals generally become carriers for up to 6 months (112, 115). Studies on the molecular characterization of *Pasteurella multocida* help elucidate the regional transmission dynamics of the disease within the One Health framework.

The clinical signs are not different from those of other respiratory diseases (dyspnea, lethargy, anorexia, pyrexia); however, edema in the mandibular region extending up to the first part of the thorax stands out. Breathing becomes difficult, and sero-foamy excretion from the nostrils and muzzle could be appreciated. In epizootics, the hyperacute manifestation of the disease is common, and sudden death occurs without clinical signs. Postmortem findings include subcutaneous edema and petechiae in regional lymph nodes, heart, and serosa. Hyperemia and congestion in the abomasum can be observed. When identifying the agent, the herd’s clinical history must be considered. Generally, there are no signs of disease other than sudden death, mainly in young animals (115). Making a differential diagnosis with other diseases, including non-infectious ones, is important. Sampling after the necropsy should include the lymph nodes, oropharyngeal exudate, and nasal exudate for the isolation of the agent. The probability of detecting *P. multocida* in blood is low, although it must be considered. The differential diagnosis must exclude pneumonic pasteurellosis. Although bacterial isolation enables serotyping, molecular tests such as PCR are more valuable. Serological studies can be helpful in carrier animals, since the high and rapid mortality in a herd does not allow the detectable development of antibodies in blood plasma (115). Control is achieved with sanitary management, elimination of positive reactors, and vaccination. The use of bacterins as a vaccination strategy has been effective in protecting *B. bubalis* calves (112, 115). Hemorrhagic septicemia in water buffalo is characterized by rapid progression, high mortality, and marked serotype-dependent virulence, which constrain early detection and effective intervention. These features support the need for prompt molecular diagnosis and vaccination strategies specifically adapted to buffalo populations. Table 1 displays the reported prevalence of hemorrhagic septicemia worldwide by region.

## 4 Hemoparasitosis and other protozoal diseases

Hemoparasitosis is one of the main limitations to the development of developing bovine livestock in tropical and subtropical countries (116). The most relevant pathogens in Latin America and the Caribbean are the protozoa *Babesia bovis* and *B. bigemina* and the rickettsia *Anaplasma marginale*. *Rhipicephalus microplus* tick is the region’s most important vector for the three hemoparasites. The coexistence of traditional cattle and buffalo herds is recognized as a potential risk to the epidemiology of bovine hemoparasitosis in the Caribbean and Latin America, given evidence that buffalo act as reservoirs for *B. bovis, B. bigemina*, and *A. marginale* (117–120). However, the role of buffalo herds in the region’s epidemiology of bovine (*Bos*) hemoparasitosis remains unclear. For example, these animals may serve as a “dilution effect” in the transmission chain of these pathogens in places where traditional cattle and buffalo coexist (118) due to their greater natural resistance to ticks (121). In the sections that follow, we will address the main epidemiological findings on hemoparasites in water buffaloes in Latin America. In addition, other protozoa of interest in the region reported in buffaloes, such as *Neospora caninum* and *Toxoplasma gondii, will be discussed.*

### 4.1 Tick infestation in buffaloes and its role as a vector of hemoparasites

**Figure 1:**
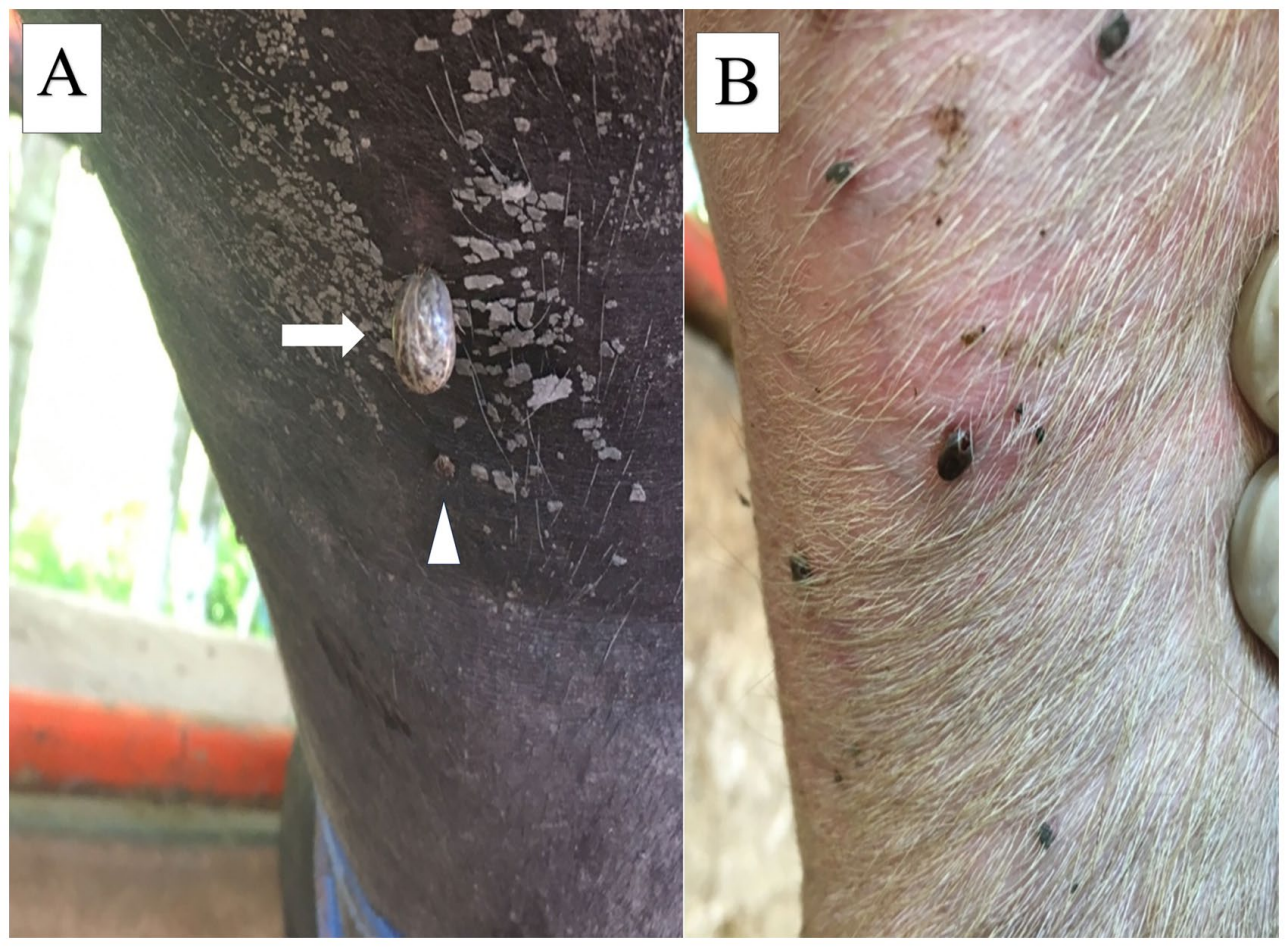
**(A)** Black water buffalo with an engorged female *Amblyomma mixtum on* the skin of the cervical region (arrow) and a small male below the female (arrowhead) in a ranch in Veracruz, Mexico. It is the most frequent tick on buffalo in Mexico. **(B)** Hind leg of an albino female water buffalo with *Amblyomma mixtum*, *ra*nch in Misantla, Veracruz, Mexico. The image is courtesy of Dr. Dora Romero Salas, Parasitology Laboratory, Faculty of Veterinary Medicine and Animal Science, Veracruzana University, Veracruz, Mexico.

Buffaloes are parasitized by ticks in most American countries where these animals are found, especially by *R. microplus*, which is endemic across most of the region’s territories (Figure 1). Infestation by *R. microplus* adult females of *R. microplus* in adult buffaloes is rare, whereas infestations in young animals are usually frequent, with high parasite loads (121). In an experimental study in Argentina, Benitez et al. (122) observed that *R. microplus* is capable of completing its entire life cycle in buffalo, concluding that buffaloes can sustain of *R. microplus* populations. These researchers also found that only 5% of the larvae that fed on buffalo survived to the teleogynous stage, whereas in bovines, the survival rate was 12%. Subsequently, Obregón et al. (121) found that ticks fed on buffaloes have the same reproductive efficiency as those fed on bovines and even disseminate *B. bovis* and *B. bigemina* transovarially for their progeny. It was found that the survival rate of *R. microplus* larvae is lower in buffalo calves than in (*Bos*) calves, showing buffaloes’ natural resistance to ticks.

**Figure 2:**
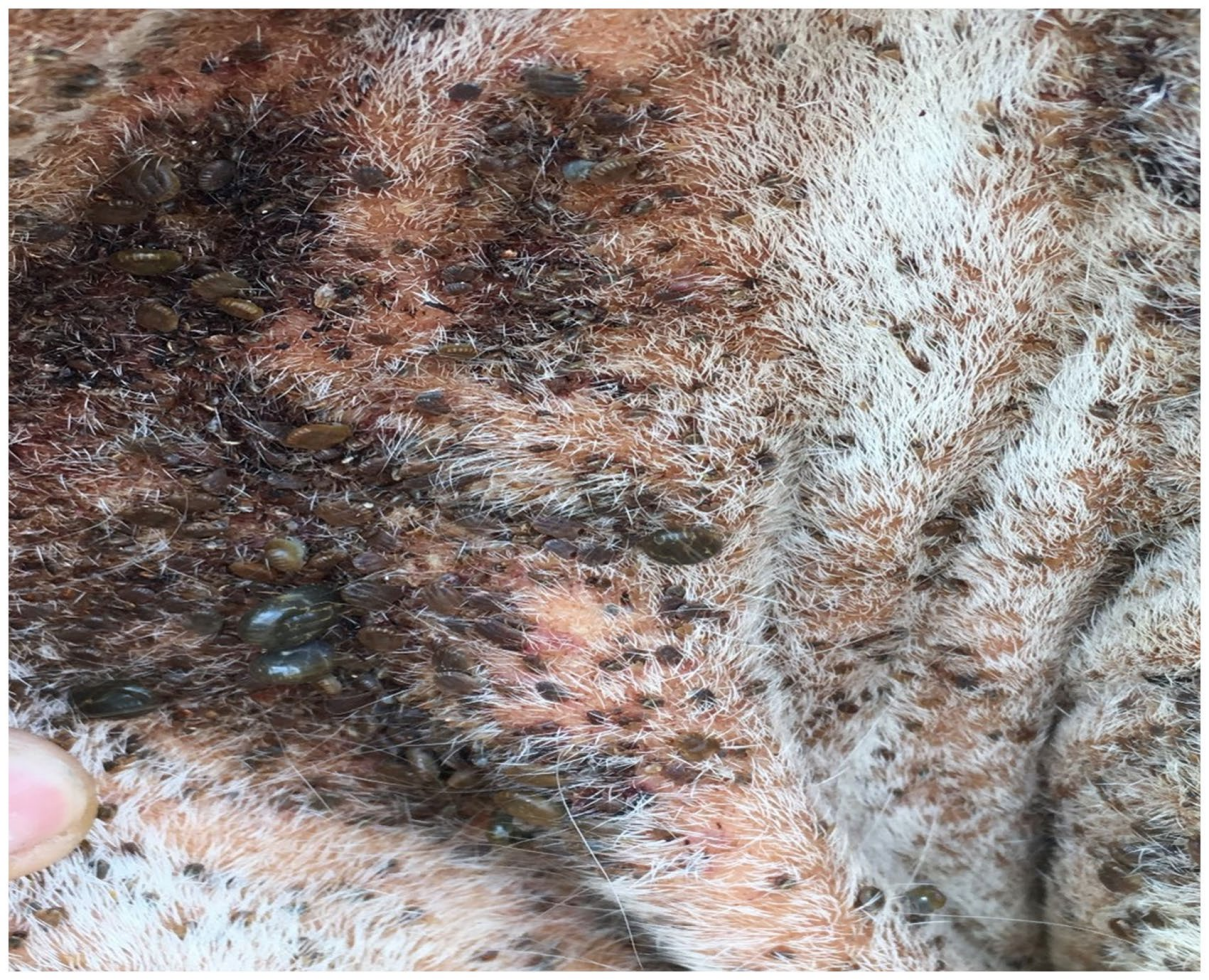
Pectoral region from an adult water buffalo with a severe mixed infestation of *Amblyomma mixtum and Rhipicephalus microplus tic*ks in different development stages at a ranch in Misantla Veracruz, Mexico. The image is courtesy of Dr. Dora Romero Salas, Parasitology Laboratory, Faculty of Veterinary Medicine and Animal Science, Veracruzana University, Veracruz, Mexico.

The water buffalo was introduced to Mexico in 1992 as a productive source of milk and meat. However, no research was conducted initially to determine which tick species began parasitizing them after their arrival until 2018, when the presence of *Amblyomma mixtum*, a neotropical tick belonging to the *Amblyomma cajennense* complex, was reported for the first time on water buffalo in Mexico (123)(Figure 1). Although in Brazil a nearby species, *Amblyomma cajennense* sensu stricto, has been recorded, the record of *A. mixtum* is important because this species is more adaptable and can be found in different habitats, from semi-arid grasslands to subtropical secondary forests, even in areas with consistently high temperatures. Additionally, *A. mixtum* has shown a strong capacity to adapt to cattle as hosts, so its presence in other bovine species, such as the water buffalo, is not surprising. The number of ticks collected at both sites confirms their presence and suggests that they have begun to form a close relationship with the water buffalo (Figure 2). However, further studies are needed to conduct systematic research to determin the impact of *A. mixtum* on water buffalo populations. This will help clarify its role in the epidemiology of certain tick-borne diseases and define the type and intensity of control measures to be implemented (123).

*B. bovis* and *B. bigemina* in *R. microplus* generate transovarian transmission, which is the main mechanism of the persistence of these protozoa in the ecosystems of Latin America and the Caribbean. Infestation of buffaloes with immature ticks can facilitate the transmission of hemoparasites, which are transmitted by different tick developmental stages; for example, *B. bovis* is transmitted only by larvae, while *B. bigemina* is transmitted by nymphs and adults (124). Nevertheless, there is still a need for more robust scientific evidence regarding the transmission of these pathogens by ticks in buffaloes and from buffaloes to cattle.

In the case of *A. marginale*, several research groups agree that it is not transmitted transovarially in ticks. Because *R. microplus* is a single-host tick, males carry out transmission, moving from host to host in search of females to mate with (125). Evidence suggests that this transmission mechanism also affects buffaloes (119). However, da Silva et al. (126) suggest that in Brazil, ticks of the *Amblyomma cajennense* and *Dermacentor nitens* complex also transmit *A. marginale* in buffaloes. Still, studies have yet to be described that prove these postulates.

### 4.2 Anaplasmosis and babesiosis

In Latin American and Caribbean countries, anaplasmosis and babesiosis are recognized as a complex of diseases called Cattle Tick Fever (or bovine parasite Tristeza in Brazil) due to their similarity in clinical symptoms and because they occur together in herds and are transmitted by the same vector. Regarding the etiological agents, *A. marginale* is a rickettsia of the Anaplasmataceae family that parasitizes the host’s erythrocytes. It is transmitted by ticks, where it multiplies in the salivary glands (125), and mechanically via blood-sucking flies, fomites, and through the placenta. As for *B. bovis* and *B. bigemina*, they are protozoa that belong to the Apicomplexa phylum (124). Protozoa of the genus Babesia carry out their asexual replication within the erythrocytes of the vertebrate host and further develop in various cell types of the ixodid vector (biological vectors) (127).

Other ruminant species can also be infected by *B. bovis*, *B. bigemina* (127), and *A. marginale* (128). However, the evidence on natural infection in these hosts and their reservoir capacity is limited, particularly regarding their role as sources of infection for transmitting pathogens from carrier buffaloes. In *A. marginale*, because it can be transmitted by blood-sucking insects, the range of hosts that can be infected is wide (128). Even so, biological transmission by ticks is the main form of distribution (125), more efficient than transmission by flies (129).

Water buffaloes can be carriers of *A. marginale* in the Caribbean and Latin America (119, 130). Few studies have been developed on the incidence of anaplasmosis in buffaloes, but it is known that its prevalence is lower than in cattle. In Brazil, da Silva et al. (130) analyzed 500 samples from 16 provinces and found a seroprevalence of 49%, while the pathogen was only detected molecularly in 5.4% of the animals. Obregón et al. (118) found molecular and serological prevalences of 51 and 60% in buffaloes from the four western provinces of Cuba, respectively. More recently, in Brazil, using qPCR, *A. marginale* was detected as being directed with the msp1β gene in 49.1% of tick samples (131).

There is consensus that, even when infected with *A. marginale*, buffaloes rarely manifest the disease clinically (118, 130). None of the available epidemiological studies reported clinical signs of anaplasmosis in buffaloes, nor did reports from of consulted breeders and veterinarians. As far as we know, only one study has reported clinical signs of anaplasmosis in buffaloes in Brazil (132). Regarding cross-dispersion of *A. marginale* among cattle and buffalo, da Silva et al. (126) in Brazil found *A. marginale* strains present in buffaloes, that were phylogenetically associated with those reported in the herd. These authors reported an isolate of *A. marginale* from *Amblyomma* ticks, which was found only in buffaloes. However, the role of *Amblyomma* spp. as a vectors of *A. marginale*, as well as the presence of specific strains in buffaloes, is an issue that should be studied in greater depth, preferably through experimental assays. In addition, Obregón et al. (119) reported, in a cohort study in Cuba, that buffaloes and bovines cohabiting the same pastures were infected with the same strains. The possibility that *A. marginale* strains had entered with the imported buffaloes was even ruled out since these came mainly from Australia, where a single *A. marginale* genotype has been reported, which has not been found in Cuba.

**Figure 3:**
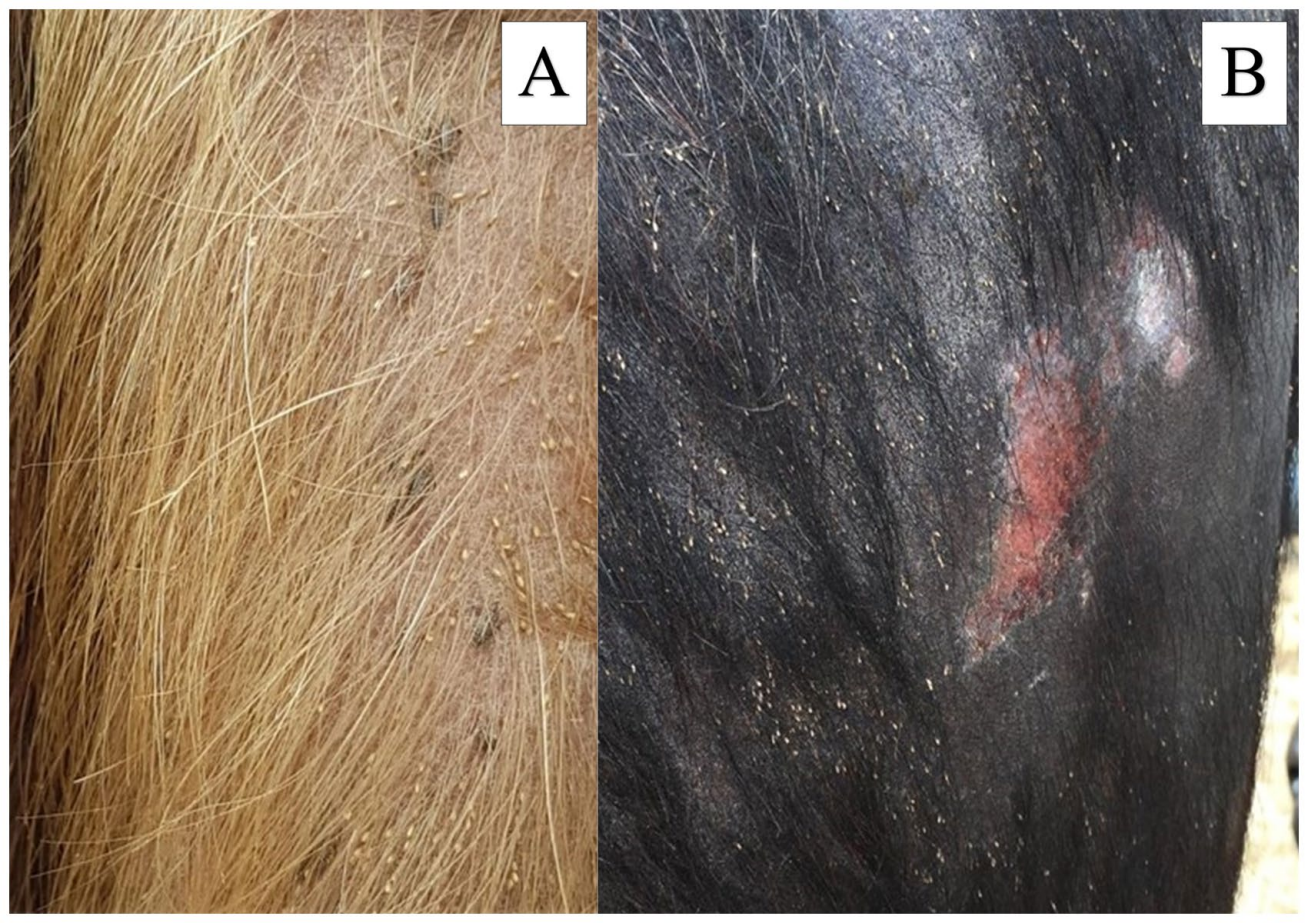
**(A)** Flank region from an albino female water buffalo, with severe infestation by *Haematopinus tuberculatus lic*e of a ranch in Medellín Veracruz, Mexico. **(B)** Calve of water buffalo with infestation by *Haematopinus tuberculatus lic*e and associated lesions. The image is courtesy of Dr. Dora Romero Salas, Parasitology Laboratory, Faculty of Veterinary Medicine and Animal Science, Veracruzana University, Veracruz, Mexico.

It is estimated that the *Haematobia irritans* and *Stomoxys calcitrans* flies may also contribute to the transmission of this pathogen in buffaloes. Furthermore, in a study focusing on *Haematopinus tuberculatus* lice on buffaloes in Brazil, they were found to be infected with *A. marginale*, identifying this louse as a possible vector (133). This fact is of greater significance because these lice are the main ectoparasites affecting buffaloes in several countries (Figure 3). In Veracruz, Mexico, blood samples of this louse collected from buffaloes were analyzed, and 70% were infected with *A. marginale* (134). More recently, in Veracruz and Tabasco, Mexico, 233 blood samples from water buffalo were evaluated to detect *Babesia* and *Anaplasma* spp., and where at least one of these hemoparasites was detected in 45% (105/233) (135). Future studies on the subject should characterize the vectorial capacity of this ectoparasite within the *A. marginale* transmission chain.

The host range in America is reduced for *B. bovis* and *B. bigemina* since their only vector is *R. microplus*, which is selective for traditional cattle. *B. bigemina* is the most frequent species, causing mortality rates of up to 30% in untreated animals. However, *B. bovis* is the most virulent species, with 70–80% mortality rates, including hematological and neurological lesions. Water buffaloes are recognized as hosts for *B. bovis* and *B. bigemina* (120, 136); In a study in Southern Thailand, *B. bigemina* and *B. bovis* were detected with 23.81 and 5.6%, respectively by PCR (137). However, buffaloes are resistant to protozoa, and infected animals are generally carriers, while clinical pictures are rare and milder than in cattle (138). The prevalence of these pathogens in buffaloes varies across Brazil, Argentina, Mexico, and Cuba. It is inferred that its prevalence depends on local epidemiological factors, especially the population dynamics of *R. microplus* (117, 118).

The findings of Romero-Salas et al. (117) in Mexico suggest that water buffaloes can eliminate *Babesia* spp., possibly due to more effective immune response mechanisms than in *Bos* bovines. Furthermore, this study found that the prevalence of *B. bovis* and *B. bigemina* in buffaloes is higher when they cohabit with infected cattle. In Colombia, the presence of Babesia spp. in cattle and buffalo has been determined to be influenced by seasonal factors, host characteristics, and vector traits. Studies indicate a higher prevalence of *B. bigemina* in cattle (Bos) and of *B. bovis* in buffalo (139). Comparable findings have been reported in Brazil and Argentina (140, 141). Taken together, these data suggest that buffalo populations in the Americas and the Caribbean have a higher frequency of *B. bovis* compared to traditional cattle. Table 1 shows the reported prevalence of anaplasmosis and babesiosis worldwide by region.

Overall, water buffaloes in the Americas act primarily as asymptomatic carriers of *Anaplasma marginale* and *Babesia* spp., with prevalence and transmission strongly modulated by vector ecology and cohabitation with cattle. The frequent discordance between molecular detection and clinical expression, together with evidence of shared straits across host species, indicates that cattle-based epidemiological assumptions may not fully capture the dynamics of these hemoparasites in buffalo populations.

### 4.3 Theileriosis

The genus Theileria is a hemoparasite that causes significant losses in cattle (genus Bos) in several regions of the world. Different species of the genus *Theileria* cause theileriosis (120). *Theileria* species are organized into two groups. The first group includes *T. parva* and *T. annulata*, which cause lymphoproliferative theileriosis in cattle. The species *T. annulata* causes tropical theileriosis, which affects cattle (*Bos*) and buffalo, and is documented in southern Europe, North Africa, the Middle East, India, and southern Russia and China. Specifically in Egypt, El-Deeb and Younis (142) found that *T. annulata* is associated with anemia in water buffaloes.

The other Theileria group is genetically diverse, including species whose taxonomic classification is still under debate and causes bovine non-lymphoproliferative theileriosis. This includes *T. sergenti*, *T. buffeli*, and *T. orientalis* (143). The *T. buffeli*/*orientalis* group affects traditional cattle and buffalo. In Thailand, Altangerel et al. (143) analyzed the *T. orientalis* genotypes in cattle and buffaloes, concluding that buffaloes act as a reservoir of these genotypes in that country. Later, Elsify et al. (144) reported the *T. orientalis* type 2 genotype in buffaloes in Egypt, which is considered a virulent genotype and therefore, of veterinary and economic interest. Fatal cases of *T. orientalis* infection in buffaloes on a dairy farm in India were also recently reported (145); In another study, the prevalence of *T. orientalis* in Southern Thailand was 29.37%, and it was associated with poor body condition in young buffalo aged 1 to 5 years (137). The *T. buffeli* clade exhibits the greatest genetic diversity, with 13 genotypes, and is distributed across the major continents, where it can infect cattle, African buffalo (*Syncerus caffer*), and water buffalo (133). Members of this clade have been assigned different species names, such as *T. buffeli, T. orientalis*, and *T. sergenti*. However, it has been proposed that they all belong to a single species called *T. buffeli*. Initially, this species originated from the Asian water buffalo in a zone where *T. annulata* did not exist (146).

Bovine (*Bos*) theileriosis is not frequent in Latin America and has not been reported in buffaloes. However, a 2016 study, in the Amazon region of Brazil, molecularly analyzed *Theileria* strains in 13 animals (n = 308) from three farms; 11 of them from a farm with a history of lymphoproliferative disease. Analyses revealed that the strains were genetically related to *T. buffeli* (147). More recently, an epidemiological study in Maranhão in northeastern Brazil detected an animal positive for *Theileria* spp. genetically related to the *T. buffeli*/*orientalis* group. These recent discoveries suggest that the epidemiological study of this disease in America should be further deepened, specifically in buffaloes, which have introduced only recently in most Latin American countries. Taken together, the evidence indicates that water buffalo plays a relevant epidemiological role in the maintenance and circulation of *Theileria* spp., particularly within the *T. buffeli/orientalis* complex, with reservoir potential and variable clinical impact. The emergence of genetically related strains in recently induced buffalo populations highlights gaps in regional surveillance and suggests that current epidemiological assumptions based on cattle may underestimated the risk and dynamics of theileriosis in buffalo. Table 1 shows the reported prevalence of Theileriosis worldwide by region.

### 4.4 Trypanosomiasis

Trypanosomes are protozoan parasites of the Trypanosomatidae family, which can infect domestic animals, including cattle and buffalo. Two modes of transmission of *Trypanosoma vivax* are described: cyclical, through tsetse flies, which occurs only in Africa, and mechanical, through contaminated fomites or through blood-sucking insects such as horseflies, (*Stomoxys calcitrans*, *and Haematobia irritans*), which occurs in Africa, South America, Asia, and other regions (148).

*Trypanosoma evansi* is the predominant pathogen in cases of “surra,” a hemoparasitosis that impacts several species, encompassing camelids, equines, buffaloes, and other mammals. In buffalo, surra is a chronic disease characterized by progressive emaciation, reproductive disorders including infertility, and the occurrence of abortions, with a high infection rate in different Asian countries. It is one of the main hemoparasites in buffaloes (149). Verloo et al. (150) in North Vietnamese water buffalo found only 1.9% of water buffalo animals positive in inoculation trials; however, 22% were seropositive, indicating that *T. evansi* disease is frequent in buffaloes in that country. Meanwhile, studies in Indonesia (151) reported a higher prevalence of trypanosomes in buffaloes compared to traditional bovines in the same region, with a higher prevalence in adult animals.

In the Americas, trypanosomiasis is mainly caused by *T. vivax* and is common in river buffalo in Central and South America, resulting in significant economic losses and 22% calf mortality (152). Specifically in Venezuela, the first studies already reported trypanosomiasis in water buffaloes in the Orinoco Delta region since the 1970s. Later, García et al. (153) found 40% seroprevalence in buffaloes. Subsequently, a longitudinal study in the Venezuelan plains during the 2006–2015 period found prevalence of 23% for trypanosomiasis in carrier buffaloes. Studies conducted in Brazil reported a higher prevalence of *Trypanosoma vivax*; Serra (2024) found a prevalence of 79.31% using the iELISA technique (154),while another study, also conducted in Brazil, reported a prevalence of 38% using real-time PCR (155). However, during a prolonged drought period in Venezuelan Llanos, a clinical trypanosomiasis event occurred, affecting more than 30% of the herd. The sick animals presented anemia and neurological disorders, with lethargy and death of 7% (156).

In Brazil, the presence of *T. vivax* has been detected in cattle from various states, including Amapá, Pará, Mato Grosso do Sul, Mato Grosso, Maranhão, Paraíba, Minas Gerais, Tocantins, São Paulo, Pernambuco, Rio Grande do Sul, Goiás, and Alagoass. This study was carried out in seasonally flooded pasture areas in Pará, in cattle (*Bos*) and buffaloes, reported prevalences of *T. vivax* of 59.6 and 44.3%, respectively, and molecularly confirmed the presence of a single *T. vivax* genotype in the region. These results highlight the need for systematic studies to characterize the epidemiology of *T. vivax* in Brazil, including cattle and buffaloes (152). Taken together, the high seroprevalence, frequent carrier status, and episodic clinical outbreaks of trypanosomiasis in water buffalo indicate a complex epidemiological pattern shaped by vector dynamics and environmental stressors. These characteristics suggest that buffalo-specific surveillance and control strategies are required, as cattle-based assumptions may underestimate the persistence and impact of *Trypanosoma* spp. in buffalo populations. Table 1 shows the reported prevalence of Trypanosomiasis worldwide by region.

### 4.5 Neosporosis

*Neospora caninum* is an obligate intracellular parasite, a protozoan of the Apicomplexa phylum. It causes abortions in cattle and buffalo throughout the world (157). It is possible to obtain viable N. caninum from naturally infected buffalo tissues, demonstrating that this species acts as an intermediate host for the parasite (158, 159). Contact with dogs was linked to buffalo infection in Pakistan, suggesting they could play a role in contaminating food intended for these animals (160). Buffalo neosporosis is economically significant in various nations, including India, Brazil, Vietnam and Italy (161). Although there is a well-established relationship between *Neospora caninum* and abortions in cattle (Bos), this situation occurs less frequently in water buffalo. Studies indicate that the inflammatory response in pregnant buffalo infected with *N. caninum* effectively controls the infection and, in most cases, prevents abortion (162); even in aborted fetuses, lesions such as encephalitis and myocarditis have been found (163). The prevalence of *N. caninum* in water buffalo varies between countries; for example, using the same diagnostic method (IFAT), it has been reported at 34.6% in Italy (163), 64% in São Paulo, Brazil (164), 58.8% in Egypt (165), Turkey 12% (166) and 64% in Argentina (167). However, a Peruvian Amazon study found low antibody seroprevalence in buffaloes (168).

Risk factors associated with *Neospora caninum* and its prevalence in water buffalo were reported in six ranches in central and southern Veracruz, Mexico; 543 buffalo sera were evaluated against *N. caninum* by ELISA, with 44.8% positive. The results indicate that older buffalo (≥7 years) had a higher seroprevalence (62.3%). Furthermore, buffalo associated with livestock had a higher prevalence (47.6%) than those without such contact (36.8%). This is important information for enforcing prophylactic measures on buffalo farms (169).

Also, in Veracruz, Mexico, the prevalence of *Neospora caninum* in two farms with a buffalo/bovine system was determined by collecting blood samples from the entire buffalo, bovine, and canine populations, and testing them by ELISA and immunofluorescence tests. 45.6% of the bovines presented antibodies against *N. caninum*. The buffaloes had a higher seroprevalence of 71.6%. Regarding dogs, 66.6% were positive. It was verified that water buffalo have a higher antibody level when they share space with cattle and dogs (170). Taken together, the high seroprevalence of *Neospora caninum* in water buffaloes, combined with the low incidence of abortion, indicates a predominantly subclinical infection with effective host control. These features support a primary reservoir role for buffaloes and suggest that epidemiological inferences based solely on cattle may not accurately reflect infection dynamics in this species. Table 1 shows the reported prevalence of Neosporosis worldwide by region.

### 4.6 Toxoplasmosis

*Toxoplasma gondii* is an intracellular protozoan of the phylum Apicomplexa that causes Toxoplasmosis; it is considered a zoonosis that can cause lesions and abortion in humans (171). Both buffalo and cattle are considered resistant to developing clinical Toxoplasmosis, and to date, no reliable case of isolation of viable *T. gondii* in buffalo meat has been documented (172); even so, the presence of the infection in buffalo has epidemiological relevance, since it implies a potential risk of transmission to humans (173).

In Turkey, *Toxoplasma gondii* has been detected in 21% of buffalo milk samples (166). In America, in the state of Veracruz, Mexico, researchers (173) determined the prevalence of *T. gondii* in 339 buffaloes. They researched the association between seroprevalence and the characteristics of buffaloes and their environment. Their results indicated that buffaloes in that area have a relatively high seroprevalence (48.7%) of *T. gondii* infection. Studies conducted in Brazil reported lower seroprevalence of this pathogen in buffaloes than in cattle. Still, seroprevalences of 35.5% were recently reported in the northern part of Brazil and 27.2% in the south (174). Similarly, in Argentina, antibodies against *T. gondii* were evidenced in 25.4% of buffaloes (102).

In the Caribbean region, a 2011 study of buffaloes in Trinidad and Tobago found a 7.8% seroprevalence for antibodies against *T. gondii*. This study was the first documentation of toxoplasmosis in that country, and the authors concluded that Toxoplasmosis exists in buffaloes on large farms in Trinidad and Tobago (175). In Cuba, Armas et al. (176) conducted a validation study using 400 buffalo sera and found 221 (55.3%) with anti-Toxoplasma antibodies by i/ELISA, confirming the results with latex agglutination in 99.8% of cases. This was the first finding of *T. gondii* seroprevalence in buffaloes in Cuba. Buffaloes are generally considered to have high resistance to toxoplasmosis; in fact, epidemiological studies have identified only antibodies indicating natural exposure to *T. gondii*. However, factors such as buffalo age, poor farm hygiene, and the presence of cats have been identified as increasing the risk of *T. gondii* infection in these animals. Furthermore, it is important to note that improperly frozen buffalo meat could pose a potential infection risk to humans, as could raw buffalo milk (162). The reported prevalence of Toxoplasmosis by regions worldwide is shown in Table 1.

## 5 Final considerations

Water buffalo is a livestock species of great importance globally. Moreover, these animals are known for their ruggedness and adaptability to tropical climates. However, they can also suffer from various infectious and parasitic diseases, which can negatively affect their productive performance, even though many of these diseases do not manifest themselves clinically in buffaloes, or buffaloes present less acute clinical pictures than traditional domestic cattle. From the animal health point of view, the recent introduction and expansion of buffalo herds to Latin American, South American and Caribbean countries represents an important factor to be considered by veterinary services, especially in health surveillance systems and fight and control plans for diseases of major economic importance, within a One Health framework. It should be taken into account that the introduction of buffaloes in a cattle-raising region also implies introducing a new factor in the transmission chain and the reservoir of many of the pathogens that affect bovines (*Bos*), given that the reservoir of a pathogen capable of infecting multiple species may consist of one or more populations that maintain epidemiological connections with each other, within which the pathogen can persist continuously and be transmitted to the susceptible population (177).

Among the factors to consider is the possible role that buffaloes may have in each epidemiological process, according to their characteristics of resistance or susceptibility to a specific pathogen and its vectors. Additionally, how the maintenance of the infection is related to the breeding system and the behavior (e.g., reproductive, feeding, hygienic) of the buffaloes in each context must be analyzed. For example, in Cuba, bovine brucellosis and tuberculosis have been controlled diseases in cattle for several decades; however, the introduction of buffalo herds and their extensive breeding in some coastal regions of the country was associated with an increase in the prevalence of these pathogens. It was impossible to monitor these pathogens in free-living buffaloes, which also moved extensively in search of food, often entering the grazing areas of cattle herds in neighboring farms.

Regarding hemoparasites, let us use the situation of buffalo calves in Cuba as an example. Due to the seasonal reproductive behavior of buffaloes, 70% of births occur between August–October. Hence, the calves are between three and 7 months old during the country’s dry season (January–April). Therefore, in this phase, the animals face the low availability of pastures, especially in stabled rearing conditions, which frequently affects body condition and immunity. Coincidentally, there is also a stationary increase in the population of *R. microplus* ticks in this period. Hence, the combination of these factors leads to the registration of a high infestation by ticks in buffalo calves between three and 12 months of age, with an elevated prevalence of *A. marginale*, *B. bovis*, and *B. bigemina*.

## 6 Conclusion

The reviewed literature and findings reveal a growing epidemiological trend of water buffalo’s increasing involvement in the circulation of infectious diseases in mixed livestock systems. Water buffalo can act as reservoirs and sources of interspecific transmission, given their high prevalence of subclinical infections and cohabitation with cattle. These findings highlight the need to include this species in surveillance and health management programs. However, gaps remain in research on specific epidemiology, and systematic studies are lacking. Current knowledge remains fragmented, focused on specific pathogens or geographically limited, and is frequently extrapolated from cattle without fully considering the biological and epidemiological characteristics of this species.

For all the reviewed findings, we believe that the surveillance and sanitary management of infectious and parasitic diseases in conditions of coexistence of cattle and buffaloes must be a holistic process, where each factor must be analyzed and addressed according to its implications and relevance for the control of the group of diseases present in each region. The findings summarized here highlight the epidemiological relevance of the water buffalo as a host and reservoir for multiple bacterial agents and hemoparasites, especially when integrated with cattle in mixed production systems, which can be a significant risk for infections in humans by zoonotic agents, within the framework of the One Health approach. These results reinforce the need to incorporate this species into health surveillance and disease control programs. Furthermore, further research is recommended on the dynamics of host-vector-pathogen transmission, the impact of management systems, and the molecular characterization of infectious agents associated with the expansion of buffalo production in order to evaluate public health risks.
